# Pregnancy-lactation cycle: how to use imaging methods for breast evaluation

**DOI:** 10.1590/0100-3984.2019.0071

**Published:** 2020

**Authors:** Carlos Henrique de Sousa Rosas, Ana Carolina de Ataíde Góes, Laís Martinho Saltão, Adriana Michiko da Silva Tanaka, Elvira Ferreira Marques, Almir Galvão Vieira Bitencourt

**Affiliations:** 1 A.C.Camargo Cancer Center, Departamento de Imagem, São Paulo, SP, Brazil.; 2 Amaral Costa Medicina Diagnóstica, Belém, PA, Brazil.

**Keywords:** Pregnancy, Lactation, Breast, Imaging diagnosis, Gestação, Lactação, Mama, Diagnóstico por imagem

## Abstract

Pregnancy and lactation constitute states of intense hormonal variation with secretory and structural changes in the breast parenchyma. These changes translate into important features on breast imaging, as well as the emergence of specific benign and malignant lesions. This literature review aims to discuss the safety of the use of breast imaging methods (mammography, ultrasound, and magnetic resonance imaging) during the pregnancy-lactation cycle, and to present the expected physiological changes and imaging appearance of the main breast diseases that may occur in this period, such as galactocele, lactating adenoma, fibroadenoma, puerperal mastitis, and pregnancy-associated breast cancer.

## Abstract (pt)

A gravidez e a lactação são estados de intensa variação hormonal e mudanças estruturais e secretórias no parênquima mamário. Essas alterações se traduzem em características importantes nos aspectos de imagem da mama, bem como propiciam o surgimento de lesões benignas e malignas específicas. A presente revisão de literatura tem por objetivo discutir a segurança da utilização dos métodos imagem da mama (mamografia, ultrassonografia e ressonância magnética) durante o ciclo gravídico-lactacional e apresentar as alterações fisiológicas esperadas e os aspectos de imagem das principais doenças mamárias que podem ocorrer nesse período, como galactocele, adenoma lactacional, fibroadenoma, mastites puerperais e câncer de mama relacionado à gestação.

## INTRODUCTION

Pregnancy and lactation are biological states that induce visible changes in the mammary glands in response to hormonal stimuli^(1,2)^. Clinically, these physiological changes result in an increase in the volume, firmness, and nodularity of the breasts, making physical examination more difficult^(2,3)^. In this context, imaging tests become important as complementary diagnostic methods. Imaging methods also imply difficulties, since changes observed on breast imaging during these periods can simulate the presence of some diseases or impair the evaluation of pre-existing conditions^(3)^. Thus, these physiological changes in the breast make the clinical and radiological evaluation of these patients challenging^(4)^.

This article reviews the use of breast imaging methods during pregnancy and lactation and discusses the expected physiological changes and the main breast diseases found in these periods.

## PHYSIOLOGICAL CHANGES EXPECTED DURING PREGNANCY AND LACTATION

Breast changes during pregnancy and lactation reflect changes in the serum levels of estrogen, progesterone, and prolactin^(3-6)^. Under the influence of increasing levels of estrogen, beginning in the first trimester of pregnancy, there is ductal growth and proliferation and, to a lesser extent, alveolar-lobular growth^(3)^. In this phase, there is an expansion of glandular tissue and involution of adipose tissue^(2)^, in addition to vascular proliferation and increased blood flow^(5)^. During the second and third trimesters of pregnancy, progesterone causes lobular hyperplasia and involution of the fibrofatty stroma^(3,5)^. After delivery, the levels of estrogen and progesterone decrease, unblocking prolactin, which, when associated with the release of oxytocin, stimulates a secretory state in the breast tissue^(7)^.

This conversion, from a proliferative state during pregnancy to a secretory state during lactation, is called lactogenesis^(3)^. During lactation, the hormone prolactin predominates and is responsible for milk production^(5)^.

## IMAGING METHODS

### Ultrasonography

Ultrasonography is the method of choice for initial breast evaluation during pregnancy and lactation. It can be performed at any time since it does not have ionizing radiation or require the use of contrast, and has a sensitivity of 87-100% for the diagnosis of breast lesions^(1,3-5)^. Although there is no consensus in the literature, some authors indicate ultrasound for pregnant or lactating patients who have palpable nodules for more than two weeks^(8,9)^ or associated with spontaneous bloody nipple discharge^(10)^.

On ultrasound performed during pregnancy, the non-fatty fibroglandular component of the breast parenchyma is enlarged and demonstrates diffuse hypoechogenicity in the first trimester. In the second and third trimesters, physiological changes accompanied by lobular proliferation may cause an increase in the echogenicity of the fibroglandular parenchyma. At the end of pregnancy, hypoechoic tubular structures corresponding to ducts with colostrum are formed. During lactation, the ducts become hyperechogenic because they contain milk (the adipose component), with a predominance of diffuse hyperechogenicity. A prominent ductal system and increased vascularity are observed^(1,5)^.

### Mammography

**Figure 1:**
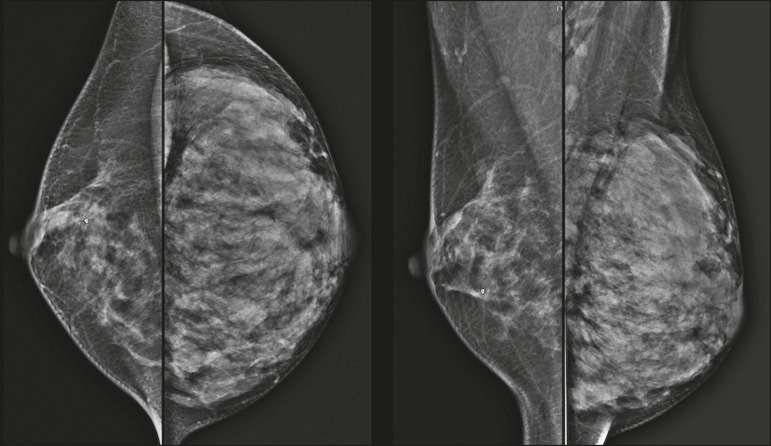
Bilateral mammography on craniocaudal and mediolateraloblique views in a lactating patient who was breastfeeding only with the left breast. Global asymmetry is observed between the breasts with an increase in volume and density in the left breast compared to the contralateral.

Mammography has a limited role during pregnancy and lactation due to the diffuse increase in density of the breast parenchyma (Figure 1)^(1,4)^, which makes it less sensitive. Although the use of ionizing radiation in mammography is worrying, the radiation dose received by the fetus, with abdominal protection, is estimated at 0.004 Gy. This dose is considered safe as fetal malformations only occur at a minimum dose of 0.05 Gy^(1,4)^. However, due to the risk of ionizing radiation to the fetus during organogenesis, mammography should be avoided in the first trimester of pregnancy^(3,6)^.

In the second and third trimesters, mammography can be performed in selected cases. Only necessary views should be obtained, and abdominal protection with a lead shield must be provided to patients^(5)^. For example, mammography can be considered in patients with clinical complaints when ultrasonography yields negative, indeterminate, or suspicious results, in lesions suspected to contain fat, or even if the biopsy of a solid lesion reveals malignancy^(4,5)^.

In lactating patients, mammography should be performed after breastfeeding or pumping, situations in which the breast density decreases^(1,4,5)^.

### Magnetic resonance imaging

Magnetic resonance imaging (MRI) of the breasts should not be performed during pregnancy. In the first trimester, MRI should be avoided due to the theoretical risk regarding the effect of the magnetic field on fetal organogenesis. In the second and third trimesters, the increase in abdominal volume makes it difficult to perform the exam in prone position. Furthermore, the use of paramagnetic contrast, which is essential for evaluating the breast parenchyma, should only be recommended when the risk-benefit ratio is clear in the evaluation of pregnant patients as intravenous gadolinium crosses the placenta^(3,4)^. Although there is no evidence that gadolinium damages the fetus, delayed fetal growth has already been reported in animal studies when it is administered in high doses^(5)^.

Although MRI can be performed during lactation, it has a limited role due to the great increase in vascularization of the breast parenchyma, which can reduce the sensitivity of the method^(3,5)^. There is no need for patients to stop breastfeeding after receiving gadolinium contrast since the dose absorbed by the newborn is 0.0004% of the injected dose, representing less than one-hundredth of the dose allowed in the infant (200 µmol/kg)^(11)^. However, if the patient wants to avoid gadolinium ingestion by the infant, breast milk should be pumped and discarded for 24 hours after gadolinium administration^(2)^.

### Percutaneous biopsies

Both fine-needle aspiration biopsy (FNAB) and large core needle biopsy can be performed during pregnancy, preferably under ultrasound guidance.

FNAB with cytological evaluation is a fast and accessible method for evaluating painful palpable cysts and collections in suspected cases of breast abscess^(12)^. It has a secondary role in the evaluation of solid nodules, and the experience of the radiologist and cytopathologist should be considered in the diagnosis as false positives may arise due to findings related to pregnancy and lactation, such as lactational hyperplasia and hyperchromia^(1,12)^.

Core biopsy is also a safe, widely available, and cost-effective method. It is considered the gold standard for tissue evaluation of breast lesions during pregnancy and lactation^(1)^. It is advantageous over FNAB as it enables the histological and immunohistochemical subtypes to be defined^(9)^. Although biopsies are usually performed under ultrasound guidance, in cases in which mammographic findings suggest microcalcifications, they can be performed under stereotactic guidance^(12)^ in early pregnancy^(9)^. The inherent risks of complications of the procedure such as hemorrhage, milk fistulae, and infection are theoretically increased in pregnant and lactating women due to the increased vascularization of the breast parenchyma, milk production, ductal dilatation, and breast trauma that accompany breastfeeding^(1)^. Some measures can be adopted to reduce the number of complications, such as pausing breastfeeding before undergoing biopsy, using thinner needles (especially to prevent milk fistulae and galactoceles), breast compression, and performing the procedure under strict asepsis^(9)^.

## MOST FREQUENT BENIGN CHANGES

Eighty percent of patients who have a palpable lump during pregnancy and lactation are diagnosed with a benign disease^(2)^, and some if them, such as lactating adenoma and galactocele, are specific of this period^(3)^. Although many breast tumors diagnosed during pregnancy and lactation may have existed previously, they manifest themselves during this period due to hormonal and physiological changes^(1)^.

### Galactocele

Galactoceles are the most common benign masses found among lactating women^(2)^. It is usually observed after breastfeeding ceases^(1)^, but it can also be seen during lactation and, less commonly, in the third trimester of pregnancy^(2)^. It usually occurs as a result of an obstructed distal duct, which causes distension of the proximal lobular segments^(3)^, presenting clinically as a palpable and painless mass^(2)^. It can occur in one or both breasts^(5)^ and can often have complications such as infection and necrosis^(6)^. The appearance on ultrasound varies depending on the amount of fat, protein, and water^(3)^, and its chronicity^(5)^. It is usually a cystic lesion (50%), whose most characteristic feature is a cyst that forms fat-fluid or thick fluid-fluid levels. Other less common forms are solid-cystic (37%) and solid (13%). When solid, it is usually circumscribed and has posterior enhancement^(7)^, however it may have irregular shape and non-circumscribed margins in some cases^(3)^. In cases of inflammation, the patient may report local pain and the lesion may have thickened walls and heterogeneous content^(7)^.

**Figure 2:**
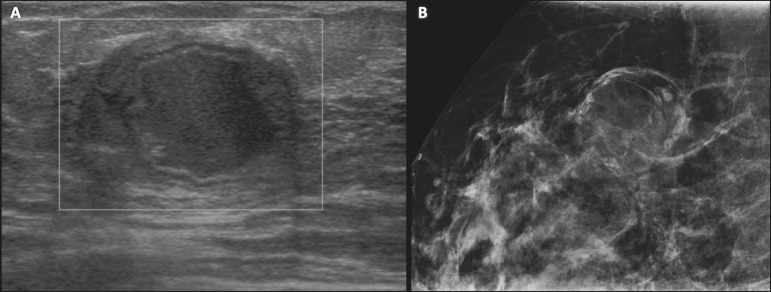
A 29-year-old lactating patient complained of a palpable mass in the right breast. Ultrasound (**A**) showed a complex cyst with hypoechoic content and thickened walls, with no significant flow on the Doppler study. Mammography (**B**) showed a nodule with peripheral calcifications and areas of fat density within it. FNAB confirmed the diagnosis of galactocele.

**Figure 3:**
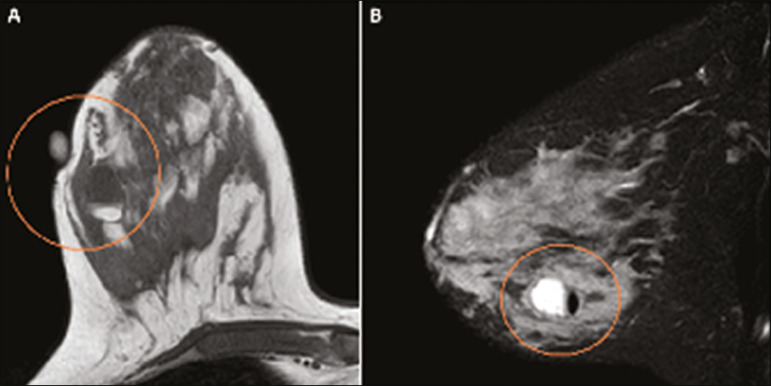
T1 (**A**) and T2 (**B**) weighted MRI images showing cyst with thin septa, heterogeneous content, and fat-fluid level, which is compatible with fat content/galactocele.

Mammography and MRI may be necessary when other diseases such as abscess and cancer are suspected as the identification of fat or fat-fluid level by these methods can confirm the diagnosis (Figures 2 and 3)^(2,3)^.

Most cases regress spontaneously^(2)^. FNAB can be performed for diagnostic or therapeutic purposes when galactocele presents as a complex mass and a differential diagnosis with other breast disorders is required, or when it is large and symptomatic^(5)^.

### Lactating adenoma

Lactating adenoma is a benign tumor related to physiological changes in the pregnancy-lactation cycle, particularly during the third trimester of pregnancy and lactation^(2)^. The true etiology of these tumors is still unknown, and there is an ongoing debate regarding whether adenoma is a new lesion or a variant of a pre-existing lesion (such as fibroadenoma and tubular adenoma) under hormonal influence^(1)^. Histologically, it consists of epithelial elements with secretory characteristics, similar to the adjacent breast parenchyma of a lactating mother^(2)^.

**Figure 4:**
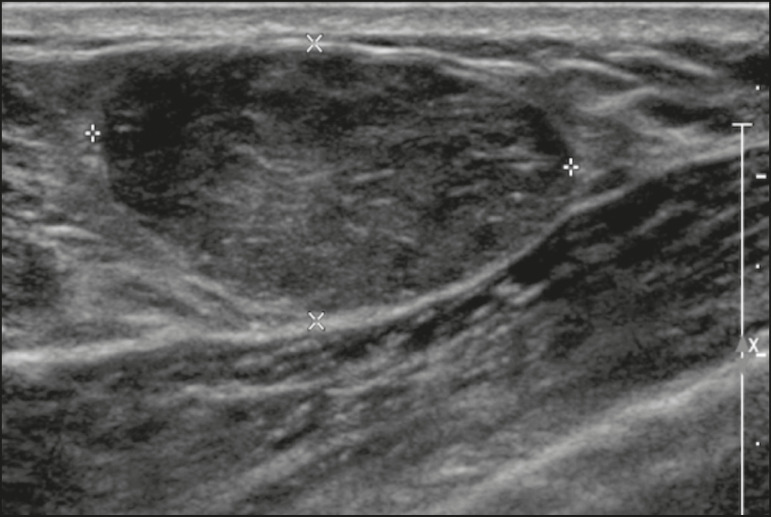
Ultrasound of the right breast showing an oval, hypoechoic, circumscribed mass, with no posterior acoustic features and with orientation parallel to the skin. Core needle biopsy confirmed the diagnosis of lactating adenoma.

The main differential diagnosis of lactating adenoma is fibroadenoma, since both can present clinically as a palpable, mobile and painless mass, with a benign appearance on ultrasonography, emphasizing that the adenoma regresses spontaneously after pregnancy or cessation of lactation^(1,3,13)^. Both lesions are indistinguishable on imaging tests, with a typical ultrasound showing an oval, circumscribed, hypoechogenic or isoechogenic lesion, with orientation parallel to the skin, showing posterior acoustic enhancement and echogenic septations (Figure 4). Doppler studies usually show that lactating adenomas are more vascular than fibroadenomas^(5)^. The presence of radiolucent areas on mammography or hyperechogenic areas on ultrasound is useful for the diagnosis of adenoma and suggests a fatty component of milk secondary to lactational hyperplasia^(1)^. In 5% of cases, adenomas can become infarcted, given the rapid growth and relative reduction in blood supply, and is characterized as a painful mass. In these cases, they can mimic a malignant lesion on ultrasonography, with an irregular, solid-cystic appearance, and posterior acoustic shadowing^(1,3,5)^. MRI also shows a nodule with circumscribed margins, presenting high or intermediate signals in T2-weighted sequences and homogeneous contrast enhancement^(7)^.

### Fibroadenoma

**Figure 5:**
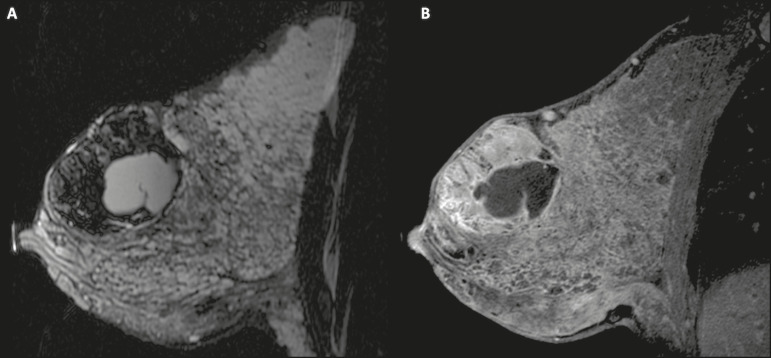
A 34-year-old lactating patient complained of a rapidly growing mass in the left breast. T2 (**A**) and post-contrast T1 (**B**) weighted MRI images showed a complex solid and cystic mass in the upper quadrants of the left breast, in the same topography of a previous biopsy compatible with fibroadenoma. A new percutaneous biopsy confirmed the diagnosis of fibroadenoma with lactational changes.

Fibroadenoma is the most common benign tumor in pregnancy and lactation, and is usually related to hormonal stimuli during these periods. Most of these tumors exist before pregnancy and their identification is facilitated by the dimensional increase resulting from the increase in hormonal levels, regressing after the interruption of breastfeeding^(1,3,5)^. Typical imaging findings are similar to those of non-pregnant non-lactating women and may have a slight increase in echogenicity on ultrasound. However, during pregnancy, they may have an atypical appearance due to secretory hyperplasia and lactational changes, with the internal accumulation of milk mimicking a galactocele (Figure 5). In these cases, ultrasonography reveals heterogeneous echogenicity, with cystic areas, sometimes forming levels, and ductal dilation^(5)^. Although rapid growth is uncommon, when it occurs, areas of infarction may appear. A clinical feature of fibroadenoma is the onset of pain at the site of a pre-existing fibroadenoma^(1)^. Similar to lactating adenoma, the presence of atypical findings on imaging studies may require anatomopathological investigation to confirm the diagnosis^(3)^.

Although they have probably benign appearance on imaging (BI-RADS 3), fibroadenomas in pregnancy and lactation should be managed in two different ways depending on the patients. Patients with masses diagnosed during pregnancy should be evaluated individually, considering risk factors and the family history. Masses with probably benign characteristics smaller than 10 mm can be followed and those with suspicious characteristics or larger than 30 mm should be biopsied, while those with dimensions between 10-30 mm must be treated individually. Patients with probably benign masses diagnosed prior to pregnancy should be monitored. If the morphological appearance remains stable with a growth rate of up to 20%, expectant management should be considered. However, if there is a growth rate greater than 20% or morphological change, a percutaneous biopsy should be performed^(12)^. Other findings that suggest the need for percutaneous biopsy are giant fibroadenomas (larger than 50 mm) and suspicious clinical findings such as skin thickening or ulceration, and papillary retraction^(14)^.

### Puerperal mastitis and abscess

Mastitis is an inflammatory process of the breast, sometimes caused by an infection. Abscess is the most common complication and is characterized as a purulent collection. Although mastitis rarely occurs during pregnancy, it is relatively common during lactation, and occurs in approximately 10% of lactating women, generally in the first six weeks after delivery^(15)^. Its pathophysiology is explained by the bacterial transmission originating from the infant’s nose and mouth to the mother through breast fissures. Other risk factors include milk stasis (as this is an excellent bacterial culture medium), duct obstruction, and breast engorgement^(4)^. The main etiologic agents are *Staphylococcus aureus* and *Streptococcus* ^(15)^. While the first causes a more localized process the second tends to manifest itself as diffuse mastitis, causing abscesses only in more advanced phases^(1)^.

Approximately 5-11% of cases of puerperal mastitis progress to an abscess^(4)^. Patients usually present phlogistic signs (pain, edema, and erythema) in the breasts, sometimes associated with systemic symptoms, such as fever, body aches, and fatigue^(15)^. It is diagnosed clinically, and imaging tests are reserved for complicated cases in which an abscess is suspected or in cases refractory to treatment^(2)^. The diagnosis of abscess can be difficult in the pre-suppurative phase, and is confused with a malignant lesion in the suppurative phase^(16)^.

Ultrasound is the imaging method of choice for complicated abscess resulting from mastitis, which is typically characterized by anechoic or hypoechoic areas with thin septations or *debris,* thickened walls, not circumscribed margins and posterior acoustic enhancement^(1)^. In the Doppler study, it usually presents peripheral vascularization^(4)^. The subacute form of mastitis may show signs of periductal inflammation, characterized by fluid collections along the subareolar ducts^(1)^. Although mammography does not have a well-established role in cases of mastitis and abscess, it may show skin thickening associated with global asymmetry or a breast mass^(5)^. The abscess may have an atypical presentation and mimic a solid or solid-cystic mass, with peripheral hypervascularization in the Doppler study, resulting in a delayed diagnosis^(5)^. The management of abscesses classically includes ultrasound-guided aspiration, sometimes with therapeutic intent, reducing the duration of the disease and promoting rapid pain relief, or for differential diagnosis in atypical cases^(15)^.

### Idiopathic granulomatous mastitis

Idiopathic granulomatous mastitis is a rare inflammatory and chronic condition of the breast^(17)^. It is found in women of childbearing age, and commonly occurs among patients in the pregnancy-lactation cycle, usually within the first six years after pregnancy^(18,19)^. Although the autoimmune hypothesis and the relationship with hyperprolactinemia have been suggested as causes^(19)^, the definitive etiology remains uncertain^(17)^. The most common clinical finding is a palpable mass, with relative preservation of the retroareolar region, which can clinically mimic inflammatory breast carcinoma. Other findings include masses and skin fistulae. Reactive lymphadenopathy may be present in up to 15% of cases^(17)^. Clinical and radiological features are variable and non-specific, making the diagnosis of this entity challenging^(20)^. Thus, investigation by anatomopathological study is decisive as it shows lobular non-caseating granulomas^(18)^. Other diseases such as tuberculosis, fungal infections, sarcoidosis and Wegener’s granulomatosis should be excluded to confirm the diagnosis^(3)^.

Although not conclusive, ultrasound is the method of choice for both initial investigation and to guide biopsy, and may reveal heterogeneous single or multiple masses^(6,21)^ with circumscribed margins and a tubular appearance. Diffuse abscess and fistula formation may also be present^(4)^. Mammographic findings are also variable and may be hidden by the high density of the breast, with focal or global asymmetry being the most common finding^(22)^. Correspondingly, focal or diffuse non-mass enhancement is the most frequent presentation on MRI, which better characterizes the disease extent^(20)^.

## PREGNANCY-ASSOCIATED BREAST CANCER

**Figure 6:**
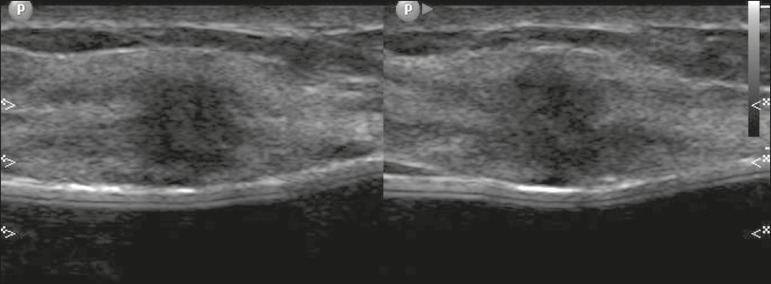
A 33-year-old patient at 27 weeks of pregnancy presented with a palpable mass in the left breast. Ultrasound showed a hypoechoic mass with irregular shape and non-circumscribed margins. A percutaneous biopsy confirmed the diagnosis of invasive ductal carcinoma.

Pregnancy-associated breast cancers refer to cancers diagnosed during pregnancy or up to one year after delivery^(3,9)^. Their incidence varies from 1:3,000 to 1:10,000 pregnancies^(9)^. Pregnancy-associated breast cancer usually has a more aggressive biological behavior, commonly presenting negative hormone (estrogen and progesterone) receptors and overexpression of human epidermal growth factor receptors type 2 (HER-2)^(3)^. The diagnosis is usually delayed due to the physiological changes that occur in the breasts during pregnancy, sometimes leading to an underestimation of the referred signs and symptoms^(23)^ and difficulty in interpreting the imaging tests. Clinically, they present as palpable masses, and ultrasound is still the best method for evaluating these lesions, allowing the differentiation of solid and cystic lesions^(4)^. They usually appear on ultrasound as masses of irregular shape and non-circumscribed margins, predominantly hypoechoic or with heterogeneous echo pattern echogenicity (Figure 6)^(9)^. Patients with suspicious lesions should undergo complementary evaluation by mammography after the first trimester of pregnancy^(9)^. Imaging findings, in these cases, are similar to those found in non-pregnant non-lactating women^(4)^.

**Figure 7:**
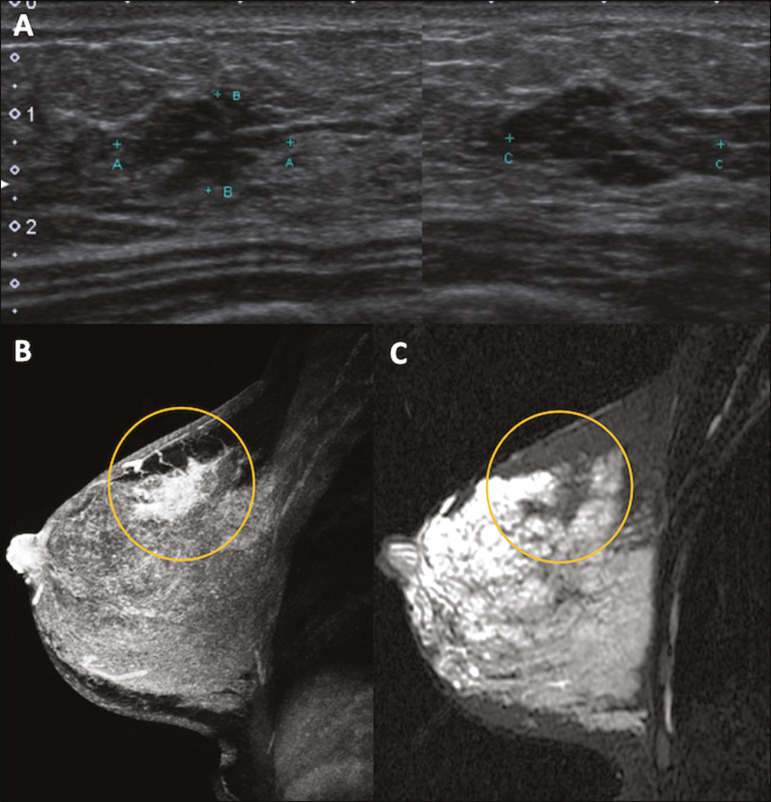
A 38-year-old patient in the sixth month of puerperium. Ultrasound (**A**) showed an irregular, hypoechoic mass with microlobulated margins. Postcontrast T1 (**B**) and T2 (**C**) weighted MRI images showed a non-mass enhancement in the upper outer quadrant of the right breast, with low signal in T2, unlike the rest of the breast parenchyma, which shows intermediate to high signal due to the lactation state.

Breast MRI studies are not performed during pregnancy, as gadolinium chelates cross the placental barrier and there are not enough studies on its effect on the fetus^(5)^. During the breastfeeding period, the American College of Radiology allows the use of gadolinium contrast^(24)^, with no need to stop breastfeeding after its injection. Although gadolinium contrast injection has been reported to be safe during breastfeeding, there is still disagreement among the authors regarding its use. In a study conducted by Myers et al.,^(25)^ preoperative MRI characterized a disease area larger than that observed on ultrasonography and mammography in 25% of patients with pregnancy-associated breast cancer, changing the surgical plan in 23% of the evaluated patients. MRI findings are similar to those found in non-pregnant patients, with masses with homogeneous, heterogeneous or peripheral enhancement, and non-mass enhancement with focal, segmental, or diffuse distribution (Figure 7)^(26)^.

## CONCLUSION

Great hormonal variation occurs in the female body during pregnancy and lactation, which causes important structural and secretory changes in the breasts. It is important that radiologists who work in the field of women’s health know about these changes, particularly regarding how to evaluate breast images, so that physiological variations are not misinterpreted and the main benign and malignant entities found in this period are diagnosed efficiently and precisely.
